# Histone modifications regulate DNA replication coupled nucleosome assembly

**DOI:** 10.1186/1756-8935-6-S1-O3

**Published:** 2013-03-18

**Authors:** Junhong Han, Qing Li, Hui Zhou, Zhiguo Zhang

**Affiliations:** 1 Department of Biochemistry and Molecular Biology, Mayo Clinic, Rochester, MN 55905, USA; 2 Current address: State Key Laboratory of Protein and Plant Gene Research and Peking-Tsinghua Center for Life Sciences, School of Life Sciences, Peking University, Beijing, China 100871

## 

DNA replication coupled nucleosome assembly plays an important role in the maintenance of genome stability and epigenetic information. In budding yeast, histone chaperones CAF-1, Rtt106 and Asf1 participate in the assembly of newly synthesized H3-H4 molecules marked by acetylation of H3 lysine 56 (H3K56ac) into nucleosomes. H3K56 acetylation is catalyzed by histone lysine acetyltransferase Rtt109-Vps75, which utilizes Asf1-H3-H4 as the substrate. A genome wide study suggests that Rtt101 functions in the same genetic pathway as Asf1 and Rtt109 to maintain genome stability. In this meeting, I will present our recent results showing that Rtt101 ubiquitylates histone H3 and histone H3 ubiquitylation regulates DNA replication coupled nucleosome assembly. In addition, we show that cells lacking this regulation are compromised in their response to DNA damage stress. Together, our studies reveal a novel mechanism whereby histone ubiquitylation regulates DNA replication coupled nucleosome assembly.

